# Effects of Illness Perception and Emotion Regulation Strategies on Posttraumatic Growth in Lung Cancer Chemotherapy Patients and Their Family Caregivers: An Actor–Partner Interdependence Model Analysis

**DOI:** 10.1155/nrp/6687304

**Published:** 2026-01-20

**Authors:** Ruihan Xiao, Linyu Zhou, Tian Xiao, Fangyi Li, Ao Tang, Biao He, Xiaoju Chen

**Affiliations:** ^1^ School of Nursing, Chengdu Medical College, Chengdu, China, cmc.edu.cn; ^2^ School of Health and Medicine, Polus International College, Chengdu, China; ^3^ Mental Health Center of West China Hospital, Sichuan University, Chengdu, China, scu.edu.cn

**Keywords:** actor–partner interdependence model, emotion regulation strategies, family caregivers, illness perception, lung cancer, posttraumatic growth

## Abstract

**Objective:**

This study aimed to investigate the dyadic relationship between illness perception, emotion regulation strategies, and posttraumatic growth in patients with lung cancer undergoing chemotherapy and their family caregivers.

**Methods:**

This study used a cross‐sectional approach to collect data from 332 pairs of lung cancer patients receiving chemotherapy and family caregivers from China. Participants completed the Brief Illness Perception Questionnaire, the Emotion Regulation Questionnaire, and the Posttraumatic Growth Inventory. The actor–partner interdependence effects were used to analyze how illness perception and emotion regulation strategies affect posttraumatic growth in the patients themselves and their family caregivers.

**Results:**

Both illness perception and emotion regulation strategies had significant actor and partner effects on posttraumatic growth in patients with lung cancer undergoing chemotherapy and their family caregivers. Emotion regulation strategies were categorized as cognitive reappraisal and expressive suppression, and patients had lower scores than caregivers for posttraumatic growth, illness perception, and expressive suppression, except for cognitive reappraisal scores, which were higher than caregivers. In patient–caregiver dyads, cognitive reappraisal was positively associated with posttraumatic growth in themselves and each other, whereas both illness perception and expressive suppression were negatively associated with posttraumatic growth.

**Conclusion:**

Reducing negative illness perceptions and expressive suppression may promote posttraumatic growth in patients with lung cancer undergoing chemotherapy and their family caregivers. Facilitating cognitive reappraisal may be useful in enhancing posttraumatic growth, which provides direction for future intervention research. Healthcare professionals should view lung cancer chemotherapy patients and their family caregivers as a whole and develop dyadic interventions.

## 1. Introduction

Lung cancer is the first malignant tumor with the highest morbidity and mortality rate in China and the world, posing a serious threat to patients’ lives [1, 2]. However, patients undergoing chemotherapy may experience a range of chemotherapy‐related adverse reactions, such as fatigue, gastrointestinal reactions, and bone marrow suppression [3]. Cancer has become a significant event impacting the whole family, and when a family member is diagnosed, relatives become cobearers of the burden, taking on nearly all caregiving tasks [4]. Family caregivers are relatives who provide care services to patients without compensation, including spouses, children, and parents [5]. The theory of shared coping [6] indicates that family members view stressful events (such as cancer) as “our problem,” and in the process of coping, they share resources and evaluations and work together to formulate strategies to manage the stressor. Therefore, during cancer treatment, it is essential to view the patient and the caregiver as a combined entity. It is crucial to focus not only on the patient’s physical and mental health but also on the caregiver’s physical and mental issues.

According to the Diagnostic and Statistical Manual of Mental Disorders, the diagnosis and treatment of lung cancer can be described as a Type A traumatic event, impacting the patient’s psychological state and quality of life [7]. Patients and caregivers are a closely connected dual entity, both facing issues such as decreased quality of life, emotional changes, and psychological distress during the treatment period [8]. Thus, the diagnosis and chemotherapy of lung cancer are traumatic experiences for both patients and caregivers. With the development of positive psychology, researchers have gradually explored the positive psychological changes in patients during the diagnosis and treatment process. Tedeschi et al. [9] first proposed the concept of posttraumatic growth (PTG) and defined it as a series of positive psychological changes that occur when an individual experiences a traumatic event, including three major aspects: changes in self‐perception, changes in relationships with others, and changes in life philosophy. Relevant studies [10, 11] have shown that increased levels of PTG can reduce patients’ self‐perceived burden, alleviate symptoms such as anxiety and depression, and improve their quality of life. At the same time, PTG also promotes caregivers’ psychological resilience, reduces emotional distress, and develops a positive life philosophy [12].

The commonsense model (CSM) of self‐regulation [13] suggests that when exposed to a health threat, individuals adjust, modify, and cope with illness cognitions and emotional responses, including cognitive and affective representations. Cognitive representations refer to the perceptions an individual develops about the characteristics of the illness, and affective representations refer to the emotional responses elicited by the health problem. The CSM may be an effective theoretical framework that explains how an individual’s perceptions of the illness and emotion regulation strategies can be used to cope with stress and health threats during the illness, which ultimately influences health behaviors and promotes levels of PTG.

Disease perception is the process of analyzing and interpreting an individual’s perception of a disease, based on known knowledge of the disease and life experience of the current disease or symptom [14]. Cancer patients who perceive more negative disease perceptions are overly preoccupied with disease symptoms, lack confidence in controlling the disease, and manifest more psychological distress [15], which may reduce the levels of PTG. Kern et al. [16] compared illness perceptions of adolescent childhood cancer survivors and their caregivers and found that caregivers showed more negative perceptions and negative emotions than patients. Disease perception not only affects cancer patients’ mental health, emotional state, and quality of life but may also impact caregivers’ emotional state and quality of life [17, 18]. Therefore, to understand the key factors influencing psychological changes in lung cancer patients and their caregivers, disease perception may be a crucial focus for researchers.

Individuals undergo emotional reactions during traumatic events, and different emotional regulation strategies may affect their ability to cope with such events [19]. The process of managing or regulating one’s emotions is referred to as emotional regulation, categorized into two strategies: cognitive reappraisal and expressive suppression [20]. Cognitive reappraisal takes place before emotions are fully activated, as the individual shifts their perspective on the event, thereby changing its emotional effect, whereas expressive suppression is a strategy focused on behavioral responses that occur after emotions have been triggered, with individuals inhibiting their emotional expression [21]. A cross‐sectional study by Li et al. [19] indicated that cognitive reappraisal helps patients reinterpret stress, reducing negative emotions, while expressive suppression hides the outward expression of internal emotions, decreases the ability to regulate emotions, and consequently reduces positive psychological changes in patients. Research by Yasaman et al. [22] on breast cancer patients revealed that employing positive emotional regulation strategies can positively influence PTG, whereas negative emotional regulation strategies harm it.

To explore the specific relationship between PTG in lung cancer chemotherapy patients and their caregivers, the actor–partner interdependence model (APIM) was used to handle dyadic data. APIM can effectively address the issue of nonindependence of dyadic data in interpersonal relationship variables. At present, an increasing number of scholars is considering patients and caregivers as a whole in their research. Wang et al. [23] employed APIM to examine the effects of family functioning and resilience on the quality of life of lung cancer patients and their caregivers, confirming the existence of interdependence between them.

To the best of our knowledge, few studies have investigated the interdependent relationships between illness perception, emotional regulation, and PTG in lung cancer patients and their caregivers. Based on the theory of CSM, this study employs the APIM to analyze the dyadic relationships between variables. It is hypothesized that the illness perception and emotional regulation strategies of lung cancer chemotherapy patients and their caregivers not only affect their own PTG (actor effects) but also mutually influence each other’s PTG (partner effects). The findings of this study are intended to offer guidance for formulating preventive measures to improve the psychological well‐being of both parties and promote PTG.

## 2. Methods

### 2.1. Study Design

This study was designed as a cross‐sectional study, with participants recruited through convenience sampling from three tertiary hospitals in Chengdu, China, from December 2023 to May 2024. The inclusion criteria for lung cancer chemotherapy patients and family caregivers are as follows: (a) patients diagnosed with lung cancer by pathological examination, (b) patients undergoing chemotherapy in the hospital, (c) both patients and caregivers are 18 years of age or older, and (d) primary family caregivers as identified by the patients. The exclusion criteria are as follows: (a) patients and caregivers with communication barriers, (b) patients and caregivers with life‐threatening illnesses, and (c) caregivers who receive compensation.

### 2.2. Sample and Recruitment

This investigation was initiated after receiving ethical approval and consent from both the hospital and the oncology department. The purpose and content of the study were explained to the participants before the survey to ensure that both the patients and their family caregivers gave informed consent and were willing to participate in the survey. If participants had any questions, investigators used standardized explanations to clarify them. Patients and caregivers completed the survey in two separate rooms without being allowed to discuss. The questionnaires were collected immediately after the participants completed the survey and checked for any missing content.

The APIMPowerR program was used to calculate the required sample size for the APIM. Considering that this study employs a distinguishable dyad model, with a power level of 0.95, a medium effect size of 0.25, and a significance level (α) of 0.05, at least 261 dyads were necessary to ensure adequate statistical power for the APIM analysis. The final number of valid questionnaires collected was 332 dyads.

### 2.3. Ethical Considerations

This study was performed in line with the principles of the Declaration of Helsinki. This study was approved by the Ethics Committee of Chengdu Medical College (2023No.109). All participants provided written informed consent.

### 2.4. Measures

#### 2.4.1. Sociodemographic and Clinical Data

Data on sociodemographic and clinical characteristics were acquired via a purpose‐designed questionnaire. The collected variables encompassed gender, age, educational attainment, employment status, residence, monthly household income per capita, time since diagnosis, lung cancer histological type, disease stage, caregiver relationship to the patient, and total weekly caregiving hours.

#### 2.4.2. PTG

Based on the Posttraumatic Growth Inventory (PTGI), originally developed by Tedeschi et al. [9], this study employed the Chinese version translated and adapted by Wang Ji et al. [24] to assess participants’ levels of PTG. The scale includes the following 5 dimensions: new possibilities, relationships with others, personal strength, self‐change, and life insights, with a total of 20 items. The scale employs a six‐point Likert scoring system (0 = not at all and 5 = very much), yielding total scores ranging from 0 to 100, with higher scores indicating greater levels of PTG. The Chinese version of PTGI has demonstrated satisfactory reliability in studies involving couples affected by gynecological cancers [25]. In this study, Cronbach’s α was 0.94 for patients and 0.93 for caregivers, indicating excellent internal consistency.

#### 2.4.3. Emotion Regulation

Emotion regulation strategies in patients and their caregivers were assessed using the Emotion Regulation Questionnaire (ERQ), originally developed by Gross et al. [20] and translated and revised by Wang Li et al. [26]. The questionnaire comprises 10 items across two dimensions: six items assessing cognitive reappraisal and four items assessing expressive suppression. Responses were recorded on a seven‐point Likert scale, ranging from 1 (“*strongly disagree*”) to 7 (“*strongly agree*”). Higher mean scores on each subscale indicate a greater tendency to employ the corresponding emotion regulation strategy. The ERQ has demonstrated satisfactory reliability and validity among Chinese adolescents and their caregivers [27]. In this study, Cronbach’s α coefficients for the cognitive reappraisal subscale were 0.85 for patients and 0.84 for caregivers, whereas those for the expressive suppression subscale were 0.88 for patients and 0.81 for caregivers, indicating good internal consistency.

#### 2.4.4. Illness Perception

Illness perception was assessed using the Brief Illness Perception Questionnaire (BIPQ), originally developed by Broadbent [28] and adjusted by Mei et al. [29]. The scale includes 8 items, each rated from 0 to 10, designed to evaluate emotional responses and cognitive representations of their illness. Higher total scores indicate a stronger perception of the illness threat and a heightened level of negative illness‐related cognition. The BIPQ has demonstrated satisfactory reliability and validity among Chinese breast cancer patients and their caregivers [30]. In this study, Cronbach’s α coefficients were 0.73 for patients and 0.71 for caregivers, indicating acceptable internal consistency.

### 2.5. Data Analysis

SPSS Version 26 (IBM Corp, Armonk, New York) and MPLUS 8.3 were used for the descriptive analysis and APIM analyses, respectively. Statistical analysis was conducted in three phases, which are described below.

In the first phase, general characteristics of lung cancer chemotherapy patients and their caregivers were described using means, standard deviations (SDs), frequencies, and percentages. Paired samples *t*‐tests were used to analyze differences in PTG, illness perception, and emotion regulation strategies between lung cancer chemotherapy patients and their caregivers. Pearson correlation analysis was conducted to test the relationships between continuous variables.

In the second phase, to empirically confirm the distinguishability between the members of the dyads based on their role, the test of complete indistinguishability was performed comparing the saturated model with the model in which actor and partner effects, intercepts, error variances, means, and variances were posed as equal. If *p* < 0.05, the two models are considered significantly different, indicating a distinguishable dyadic relationship. Conversely, if *p* > 0.05, the models are indistinguishable [31–33].

In the third phase, in order to assess the actor and partner effects of illness perception and emotion regulation strategies on PTG, we utilized MPLUS software to build several APIM structural equation models. The basic APIM was a fully saturated model. The path coefficients are reported as standardized coefficients in order to increase comparability across variables.

## 3. Results

### 3.1. Sociodemographic and Clinical Characteristics of Participants

A total of 332 dyads of lung cancer patients undergoing chemotherapy and their family caregivers were included in this study. The patients were predominantly male (69.3%); more than half were older than 60 years (60.2%); and more were still employed (43.6%). Adenocarcinoma was diagnosed in 56.9% of patients, with a predominantly Stage IV disease (77.1%), and the time since diagnosis was predominantly less than 6 months (35.8%). Among the caregivers, the majority of caregivers were female (70.2%), and most of them were in the age range of 45–59 years (41.6%); the primary caregiver was predominantly the spouse (70.2%), and 23.5% had children in the role of caregiver. More detailed information is presented in Table 1.

**Table 1:** Sociodemographic and clinical characteristics of participants (*n* = 332 dyads).

Variables	Patients	Caregiver
	*n* (%)	*n* (%)
Gender		
Male	230 (69.3)	233 (70.2)
Female	102 (30.7)	99 (29.8)
Age		
< 45	9 (2.7)	123 (37)
45–59	123 (37.0)	138 (41.6)
> 60	200 (60.2)	71 (21.4)
Education level		
Primary school and below	190 (57.2)	168 (50.6)
Junior high school	88 (26.5)	100 (30.1)
Vocational high school/high school	37 (11.1)	40 (12)
College degree or above	17 (5.1)	24 (7.2)
Employment status		
Employed	145 (43.6)	187 (56.3)
Unemployed	111 (33.4)	111 (33.4))
Retired	76 (22.9)	34 (10.2)
Residence		
City	175 (52.7)	201 (60.5)
Countryside	157 (47.3)	131 (39.5)
Per capita monthly household income (RMB)		
< 1000	37 (11.1)	
1000–3000	81 (24.4)	
3001–5000	125 (37.7)	
> 5000	89 (26.8)	
Time since diagnosis		
< 6 months	119 (35.8)	
6–12 months	78 (23.5)	
13–24 months	45 (13.6)	
> 2 years	90 (27.1)	
Lung cancer type		
Squamous cell carcinoma	103 (31)	
Adenocarcinoma	189 (56.9)	
Small‐cell carcinoma	40 (12)	
Number of chemotherapy treatments		
< 4	118 (35.5)	
4–6	107 (32.2)	
> 6	107 (32.2)	
Disease staging		
I	3 (0.9)	
II	13 (3.9)	
III	60 (18.1)	
IV	256 (77.1)	
Caregivers’ relationship with patients		
Spouse	233 (70.2)	
Daughter/son	78 (23.5)	
Parent	8 (2.4)	
Brother/sister	5 (1.5)	
Others	8 (2.4)	
Total hours of care for patients		
< 3 months		59 (17.8)
3–6 months		70 (21.1)
7–12 months		71 (21.4)
> 1 year		132 (39.8)

### 3.2. Correlation of Illness Perception, Emotion Regulation Strategies, and PTG

Paired‐sample *t*‐tests were used to compare the differences between patients and caregivers for each variable, and the specific means, SDs, and *t*‐test results are shown in Table 2. Results showed significant differences between patients and caregivers on each variable, with caregivers having higher PTG, illness perception, and expressive suppression than patients, except for lower cognitive reappraisal scores. Table 3 shows the correlation between the variables. While the rest of the variables were negatively connected, in patient–caregiver dyads, cognitive reappraisal had a positive correlation with both their and the other’s PTG.

**Table 2:** Comparison of study variables between patients with lung cancer undergoing chemotherapy and their family caregivers (*n* = 332 dyads).

Variables	Patients mean ± *SD*	Caregivers mean ± *SD*	Paired *t*	*P*
PTG	57.93 ± 13.49	64.96 ± 12.69	−16.335	< 0.001
Illness perception	44.91 ± 8.13	46.36 ± 8.46	−4.016	< 0.001
Cognitive reappraisal	23.64 ± 5.08	22.76 ± 4.98	3.823	< 0.001
Expressive suppression	14.31 ± 4.69	18.13 ± 4.57	−17.3	< 0.001

Abbreviation: PTG, posttraumatic growth.

**Table 3:** Correlations among illness perception, emotion regulation strategies, and PTG in patients with lung cancer undergoing chemotherapy and their family caregivers (*n* = 332 dyads).

	1	2	3	4	5	6	7	8
1 Patients’ PTG	1							
2 Patients’ illness perception	−0.665^∗∗^	1						
3 Patients’ cognitive reappraisal	0.666^∗∗^	−0.658^∗∗^	1					
4 Patients’ expressive suppression	−0.679^∗∗^	0.537^∗∗^	−0.659^∗∗^	1				
5 Caregivers’ PTG	0.822^∗∗^	−0.620^∗∗^	0.614^∗∗^	−0.649^∗∗^	1			
6 Caregivers’ illness perception	−0.664^∗∗^	0.685^∗∗^	−0.618^∗∗^	0.529^∗∗^	−0.693^∗∗^	1		
7 Caregivers’ cognitive reappraisal	0.664^∗∗^	−0.547^∗∗^	0.655^∗∗^	−0.540^∗∗^	0.530^∗∗^	−0.537^∗∗^	1	
8 Caregivers’ expressive suppression	−0.677^∗∗^	0.508^∗∗^	−0.536^∗∗^	0.622^∗∗^	−0.592^∗∗^	0.479^∗∗^	−0.606^∗∗^	

Abbreviation: PTG, posttraumatic growth.

^∗∗^ *P* < 0.01.

### 3.3. Test of Distinguishability

Statistically significant *χ* ^2^ differences (Δ*χ* ^2^) were found for PTG (Δ*χ* ^2^ = 20.293, *p* < 0.05). This indicates that the members of the dyads could be treated as distinguishable on the basis of their roles.

### 3.4. APIM

**Figure 1:**
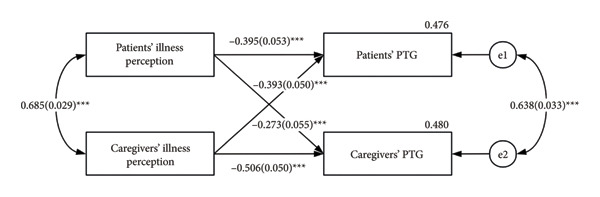
APIM for illness perception on PTG. PTG, posttraumatic growth. The estimates are standardized regression coefficients. ^∗∗∗^ *p* < 0.001.

The associations of illness perception and PTG are shown in Figure 1; both significant actor (*β* = −0.395, *p* < 0.001; *β* = −0.506, *p* < 0.001) and partner (*β* = −0.393, *p* < 0.001; *β* = −0.273, *p* < 0.001) effects were found for patients with lung cancer undergoing chemotherapy and their family caregivers. The results indicate that patients’ illness perception can negatively influence not only their own PTG but also that of their caregivers. Caregivers’ illness perception can negatively influence not only their own PTG but also that of their patients.

**Figure 2:**
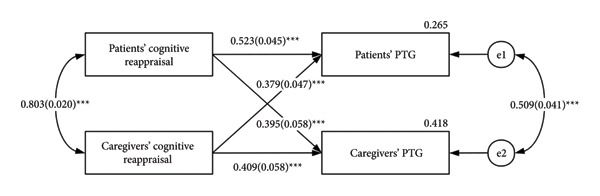
APIM for cognitive reappraisal on PTG. PTG, posttraumatic growth. The estimates are standardized regression coefficients. ^∗∗∗^ *p* < 0.001.

The associations of cognitive reappraisal and PTG are shown in Figure 2. From the actor effects, both patients’ and caregivers’ cognitive reappraisals had a positive effect on their own PTG (*β* = 0.523, *p* < 0.001; *β* = 0.409, *p* < 0.001). In terms of partner effects, there was a significant positive effect of cognitive reappraisal of patients on the PTG of caregivers (*β* = 0.395, *p* < 0.001), and the cognitive reappraisal of caregivers also had a positive effect on the PTG of patients (*β* = 0.379, *p* < 0.001).

**Figure 3:**
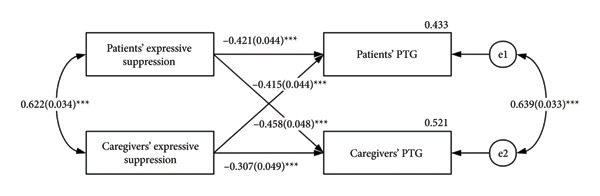
APIM for expressive suppression on PTG. PTG, posttraumatic growth. The estimates are standardized regression coefficients. ^∗∗∗^ *p* < 0.001.

The associations of expressive suppression and PTG are shown in Figure 3, with all involved actor and partner effects statistically significant for both patients and caregivers (*p* < 0.001), suggesting that expressive suppression not only directly and negatively predicts their PTG but also negatively predicts each other’s PTG.

## 4. Discussion

This was the first attempt to use APIM to examine the dyadic relationships between PTG, illness perception, and emotion regulation strategies among patients with lung cancer undergoing chemotherapy and their family caregivers. First, we identified differences in the levels of PTG, illness perception, cognitive reappraisal, and expressive suppression between patients with lung cancer undergoing chemotherapy and their family caregivers. In addition, our findings suggest that cognitive reappraisal may be a positive coping strategy that enhances PTG levels in patients and caregivers. Significant actor and partner effects were found for PTG, illness perception, cognitive reappraisal, and expressive suppression in both patients and caregivers, emphasizing the importance that health professionals should view patients and caregivers as a whole.

Our findings showed that patients had significantly lower PTG levels than their caregivers, different from the findings of Song et al. [25] on gynecological cancer couples. This may be since 70.2% of the caregivers in this study were female and 69.3% of the patients were male. In a study of adults, it was demonstrated that women reported significantly more PTG than men [34]. Furthermore, in the traditional context of Chinese culture, men are expected to show strength and stoicism, and society is more tolerant of women showing their inner vulnerability and sensibility, which promotes their reflection on their traumatic experiences, helps them to generate more insights into their lives and self‐transformation, and ultimately benefits from PTG. Therefore, based on the fact that the majority of our caregiver sample was female, caregivers may report higher levels of PTG than patients.

It is noteworthy that caregivers in the present study had significantly higher levels of illness perception than patients undergoing chemotherapy for lung cancer, in contrast to Wang et al. [30], who did not find a difference in their study of breast cancer patients and their spouses. The occurrence of such differences may be related to the burden of caregiving and the sample characteristics of the patients. First of all, when patients receive treatment, caregivers not only provide daily care, emotional support, and financial support but also work with patients to face the potential complications and adverse reactions associated with various therapeutic and nursing interventions, including symptom management during chemotherapy, wound care, and so on [35]. The additional burden of caregiving may be responsible for caregivers exhibiting more negative perceptions of illness than patients. Moreover, in the patient sample, 60.2% were older than 60 years old and had a lower level of education. Patients may have more difficulty in understanding medical terminology and treatment options when communicating information about their condition with medical staff, at which point the caregiver becomes the primary communicator and substitute decision‐maker. Information related to the disease is first communicated to the caregiver by the healthcare provider out of concern for the protection of the patient. Caregivers, therefore, receive more in‐depth information about the disease than the patient, which further increases the caregiver’s psychological burden and ultimately leads to high levels of negative illness perception.

The present study also found that caregivers were more likely to use expressive suppression as an emotion regulation strategy, whereas patients were more likely to use cognitive reappraisal as a regulation strategy. This is not well explained in the literature, as no other study has yet come to the same conclusion. This may be because, on the one hand, cancer patients and caregivers tend to avoid openly discussing cancer‐related information and emotions to protect each other from emotional distress, known as “family avoidance of communication about cancer” [36]. Caregivers tend to be more tolerant and affectionate toward patients who are already suffering from cancer, and because they do not want to add to the patient’s psychological stress, caregivers are more inclined to use expressive suppression to minimize the output of negative emotions and conceal their concerns. On the other hand, 64.2% of the patients in this study had a disease course of more than half a year; they had passed the initial stage of doubt and fear and gradually accepted the fact that they had cancer. During this period, they were willing to cooperate with anticancer treatment. The use of cognitive reappraisal as a way to regulate emotions is mainly to better cope with the disease and establish positive cognitive and psychological cues to oneself.

Our APIM analysis revealed that illness perceptions of lung cancer chemotherapy patients and caregivers influence their own PTG (actor effects). Similar to prior studies [37], we found that illness perceptions were significantly and negatively associated with PTG in lung cancer patients undergoing chemotherapy. When patients are overly concerned with the symptoms of the disease, they tend to believe that the disease will have serious consequences. Lacking confidence in controlling the disease, they experience negative psychological states such as anxiety and fear of disease progression, meaning that the more negative illness perceptions there are, the fewer positive psychological changes and lower levels of PTG the patient experiences. In addition, studies have shown that caregivers can be more concerned about the patient’s illness than the patients themselves [18]. When the negative disease information is received, the improper cognition of the patient’s disease will lead to an increase in the caregiver’s psychological stress and care burden. At the same time, overburdening can increase caregivers’ levels of depression, influence their own positive psychological resources, and decrease PTG [38]. The patient’s and caregiver’s perception of illness negatively affects each other’s PTG (partner effects). One possible explanation is that cancer puts pressure on the patient not only as an individual but also as a whole family. When individuals are overly sensitive to their perceptions of illness, it can reduce the resilience of the family as a whole to adversity [39]. This may reduce the amount of spiritual change and benefit found by both parties during the treatment process or caregiving.

The results of our APIM analyses also demonstrated that patients’ and caregivers’ cognitive reappraisals positively influenced their own PTG (actor effects). Consistent with previous studies [40], patients’ choice of positive emotion regulation strategies can help them to promptly adjust their emotional state in the face of fear and anxiety and to achieve positive psychological growth in reevaluating and understanding the illness experience. Meanwhile, for caregivers in dyadic relationships, cognitive reappraisal enables them to explore potential benefits in the caregiving process [41], such as changing poor living and eating habits. After learning more health‐related knowledge, caregivers may experience new meaning in life and achieve positive self‐change. Similarly, in terms of partner effects, the cognitive reappraisal of patients and caregivers positively affects each other’s PTG. According to the “interdependence theory” [42], there is a strong reciprocal influence between individuals in a close dyadic relationship, whereby individual cognitions, emotions, or behaviors are easily transmitted and interact with each other. When facing a stressful event, cognitive reappraisal by both parties is conducive to creating a positive dyadic coping atmosphere, and they may take the initiative to engage in self‐reflection, help each other find positive meaning from difficult situations, and explore new possibilities in life, and ultimately, both parties can achieve PTG.

The last APIM analysis showed that expressive suppression in lung cancer chemotherapy patients and caregivers negatively affected their own PTG (actor effects). When they frequently use expressive suppression as an emotional regulation strategy, they may develop social isolation, become reluctant to express their thoughts, limit contact with social support systems, and create a sense of distance from family, friends, and medical personnel. With multiple burdens and stresses that they are unable to express or release externally, they are more prone to mood swings and fall into fear of illness, increasing their own negative posttraumatic experiences. In terms of partner effects, the PTGs of lung cancer chemotherapy patients and caregivers were influenced by each other’s expression of suppression. Patients who are more inclined to use emotional suppression as a way of coping with various challenges posed by cancer not only reduce self‐expression but also create a negative caregiving experience for their caregivers. If the caregiver also adopts an expressive suppression coping style at this time, this will further aggravate the patient’s sensitive emotions. The negative response of both sides makes the other side receive no positive feedback, so they cannot get positive psychological growth from the traumatic event. The family systems theory [43], which views the family as an interconnected and interacting dynamic system in which coping strategies adopted by family members have an impact on the patient, is again supported by our study. Therefore, medical professionals should encourage lung cancer chemotherapy patients and family caregivers to express their emotions and thoughts appropriately, which can help alleviate the level of psychological distress and improve the PTG levels of both parties.

### 4.1. Limitations

Although this study provides valuable insights into the relationship between PTG and illness perceptions and emotion regulation strategies in patients with lung cancer undergoing chemotherapy and their family caregivers, there are several limitations. First, this study used a cross‐sectional research design, and in order to further explore the causal relationship between PTG, illness perception, and emotion regulation strategies, future studies will require a longitudinal research design. Second, participants were recruited from the medical oncology department only with lung cancer; therefore, the applicability of the findings to other types of cancer may be limited. Future studies could improve the generalizability of the findings by recruiting a more diverse group of cancer patients. Last, the influence of cultural background and common approach bias may introduce bias, resulting in a higher correlation between patient–caregiver PTG. In addition, the data in this study were collected through patient and caregiver self‐reports, which may lead to reporting bias and recall bias. The abovementioned limitations prompt us to engage in deeper and more rigorous consideration of quality control procedures in future research.

### 4.2. Clinical Implications

Measures centered on dyadic interventions during chemotherapy may be more effective in promoting PTG in lung cancer patients. We should emphasize the positive role of the family caregiver in the patient’s coping with the disease and develop targeted health education interventions, such as increased informational support, cognitive reframing, and active coping, to reduce negative perceptions of the disease on both sides and to foster positive perceptions [44]. At the same time, patients and caregivers are encouraged to talk about their anxieties and concerns and to use emotion regulation strategies appropriately. Reducing their inhibition of expression is conducive to healthcare professionals gaining a deeper understanding of the patient’s inner needs and helping the patient adjust their state of mind promptly, and increasing cognitive reappraisal can improve the patient’s and caregiver’s own ability to cope with traumatic events. These measures help them to summarize their experiences and gain PTG in the face of adversity. Currently, chronic disease management is increasingly emphasizing the dichotomous coping model, and in order to develop better health education content and coping strategies for patients, family caregivers, who are responsible for important caregiving tasks, should be simultaneously included in disease management strategies in the future.

## 5. Conclusion

In summary, our findings align with our hypothesis. Based on the APIM, this study not only reveals the important role of illness perception and emotion regulation strategies in influencing the PTG of patients with lung cancer undergoing chemotherapy and their family caregivers from a dichotomous perspective but also highlights the potential benefits of cognitive reappraisal in enhancing psychological growth. Both patients’ and caregivers’ illness perceptions and expressive suppression could negatively affect their own and each other’s PTGs, and cognitive reappraisal positively predicted their own and each other’s PTGs, with all actor–partner interdependence effects being significant. This study reveals that patients and caregivers are psychologically interdependent, and future dyadic research should be centered on the targeted development of dyadic interventions to improve PTG in lung cancer chemotherapy patients and their family caregivers.

## Disclosure

All authors have read and agreed to the published version of the manuscript.

## Conflicts of Interest

The authors declare no conflicts of interest.

## Author Contributions

Conception and design: Ruihan Xiao, Linyu Zhou, and Tian Xiao. Data collection: Ruihan Xiao, Linyu Zhou, and Fangyi Li. Data curation/analysis: Ruihan Xiao, Ao Tang, Fangyi Li, and Biao He. Original draft and writing–review and editing: Ruihan Xiao, Linyu Zhou, and Tian Xiao. Supervision, funding acquisition, and writing–review and editing: Xiaoju Chen. Ruihan Xiao, Linyu Zhou, and Tian Xiao have contributed equally to this work and share first authorship.

## Funding

This research was funded by Chengdu Medical College Postgraduate Innovation Fund (No. YCX2024‐01‐86) and Zigong City Philosophy and Social Science Key Research Base Health and Humanities Center 2023 Project (No. JKRWY23‐16).

## Data Availability

The data that support the findings of this study are available from the corresponding author upon reasonable request.
